# A Handbook of the Theory and Practice of Medicine

**Published:** 1891-03

**Authors:** 


*A Handbook of the Theory and Practice of Medicine.* By FREDERICK T. ROBERTS, M.D., B.Sc. Eighth Edition. London: H. K. LEWIS. 1890.

The rapid reproduction of new editions of this book is a sufficient indication of its utility and popularity. The natural tendency in the evolutional history of text-books is towards plethora and overgrowth; but this book is now in its robust manhood, and is not yet too corpulent, although it is fifteen pages larger than the last edition. It appears to be well up to date, and is worthy of the confidence that the present generation of students repose in it.

